# Multi-stage diastolic function classification algorithm by cardiac MRI demonstrates the relationship between severity of diastolic dysfunction and acute infarct size

**DOI:** 10.1186/1532-429X-11-S1-P157

**Published:** 2009-01-28

**Authors:** Sean D Pokorney, Michael Andrew Morse, Daniel C Lee, Kofo O Ogunyankin, Edwin Wu

**Affiliations:** 1 Northwestern University, Chicago, IL USA; 2 University of North Carolina, Chapel Hill, NC USA

**Keywords:** Acute Myocardial Infarction, Infarct Size, Diastolic Dysfunction, Diastolic Function, Myocardial Infarct Size

## Introduction

A previously validated multi-stage echocardiography (echo) classification algorithm for diastolic function (DF) showed that severe diastolic dysfunction independently predicts mortality after acute myocardial infarction. Currently, echo is the non-invasive imaging modality of choice for classifying diastolic dysfunction into four distinct groups based on a combination of echo parameters. However, a newer multi-stage echo algorithm has been developed that classifies patients into three diastolic function categories: Normal, Abnormal Relaxation, and Severe (which combines Pseudonormal and Restrictive patterns). This algorithm is a validated systematic approach that provides accurate diastolic function staging of patients, and it has been shown to correlate strongly with catheter derived left ventricular filling pressures. It is currently unknown whether CMR can accurately categorize the overall severity of diastolic dysfunction using a similar multi-stage approach.

## Purpose

A CMR multi-stage diastolic function classification system, adapted by substitution of CMR-derived parameters for corresponding echo parameters, can accurately classify diastolic dysfunction severity. Furthermore, applying this diastolic dysfunction algorithm can facilitate evaluation of the relationship between diastolic function and CMR infarct size.

## Methods

Subjects (n = 51) underwent CMR for characterization of left ventricular mass, left end diastolic volume, left atrial volume, ejection fraction, mitral inflow pattern, tissue velocities, and infarct size within 2.8 ± 1.4 days status post acute myocardial infarction. A subset of patients (n = 21) concurrently had echo within 2.3 ± 1.6 days of the CMR. CMR and echo data were used independently to classify subjects' diastolic function as Normal, Abnormal Relaxation, or Severe, using the multi-stage approach. The algorithm uses ejection fraction, pulmonary vein inflow, mitral inflow patterns, and deceleration time to initially categorize patients' diastolic function. If the initial parameters did not clearly delineate the appropriate classification, tissue velocity imaging, including E/E' ratios, was used to further differentiate between the three groups. Myocardial infarct sizes obtained by CMR were then compared between the resulting categories.

## Results

The severity of diastolic dysfunction using echo (n = 21) and CMR (n = 51) showed a strong relationship with acute myocardial infarction size (ANOVA, p = 0.01 and p < 0.0001 respectively). The correlation between individual parameters by echo and CMR was only fair (Table 1). However, by using the multi-stage algorithm, the overall agreement between diastolic dysfunction classifications by both modalities was good (Kappa = 0.57, p < 0.0001). CMR had 80% sensitivity and 64% specificity in detecting any diastolic dysfunction when compared to echo. In identifying severe diastolic dysfunction, CMR had 100% sensitivity and 63% specificity.

**Table 1:** Selected parameters and infarct sizes by diastolic function classification

	Echo (n = 21)			CMR (n = 51)				
	Normal (n = 14)	Abnormal Relaxation (n = 4)	Severe (n = 3)	Normal (n = 18)	Abnormal Relaxation (n = 14)	Severe (n = 19)	Echo vs CMR Correlation (r)	p-value
Ejection Fraction (%)	57.8 ± 9.1	50.9 ± 5.5	35.1 ± 9.4	51.2 ± 5.9	47.1 ± 6.6	36.4 ± 10.8	0.63	0.004
Mitral E/A	1.29 ± 0.48	0.93 ± 0.71	1.62 ± 1.45	1.22 ± 0.33	1.20 ± 0.72	1.43 ± 0.77	0.34	0.090
Mitral E DT (msec)	186.6 ± 31.1	216.3 ± 37.2	157.3 ± 31.7	188.6 ± 32.2	215.2 ± 53.0	157.7 ± 65.1	0.08	0.702
TVI E' (lateral) mm/s	10.9 ± 3.3	6.7 ± 2.1	7.3 ± 1.5	6.5 ± 2.0	6.4 ± 1.3	3.9 ± 2.2	0.83	0.002
E/E' (lateral)	8.0 ± 3.2	7.8 ± 3.3	10.4 ± 1.5	7.4 ± 2.8	7.5 ± 2.0	12.3 ± 6.4	0.70	0.020
LV Mass/Height Index (g/m^2.7)	42.0 ± 10.8	52.4 ± 23.9	51.9 ± 8.7	49.0 ± 7.0	62.1 ± 17.9	62.1 ± 17.1	0.36	0.060

Values are mean ± SD; Mitral E DT = Mitral inflow early diastolic deceleration time; TVI E' = Early diastolic tissue velocity; E/E' = ratio of early mitral inflow to early diastolic tissue velocity.

## Conclusion

**Figure 1:**
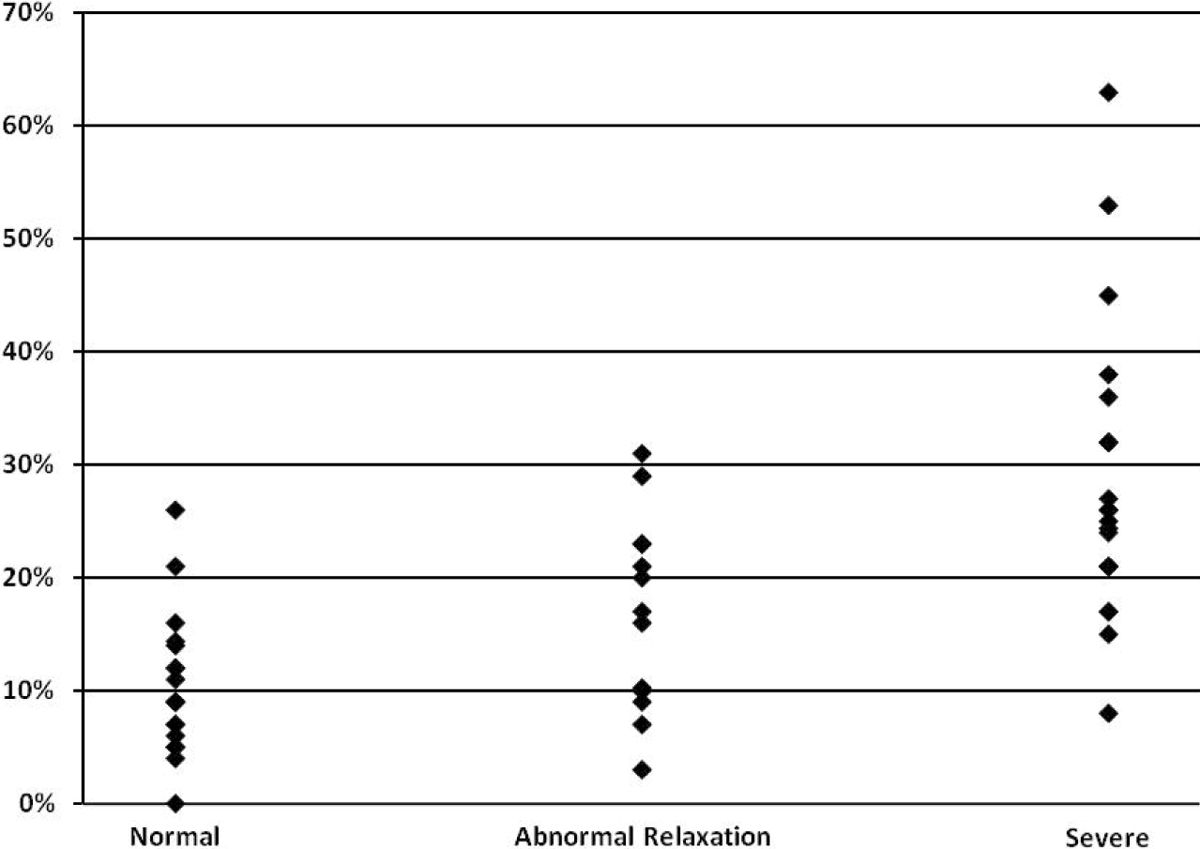
**MRI infarct size (% of LV) grouped by diastolic function**.

Correlation of single parameters of diastolic dysfunction between echocardiography and CMR is only fair. This lack of agreement is likely related to the temporal separation between the imaging studies and the rapidly evolving changes following acute myocardial infarction. However, using the multi-stage classification algorithm provides improved agreement, and this system is very sensitive for diagnosing diastolic dysfunction, especially when severe. Lastly, there is strong association between acute myocardial infarct size and severity of diastolic dysfunction (Figure 1).

